# Meet the Section Editor

**DOI:** 10.2174/1570159X2202231015185700

**Published:** 2023-11-28

**Authors:** Margarete Dulce Bagatini

**Affiliations:** 1 Department of Biological Sciences, Universidade Federal da Fronteira Sul, Chapecó, Brazil

Dr. Margarete Dulce Bagatini is an associate professor at the Federal University of Fronteira Sul/Brazil. She has a degree in Pharmacy and Ph.D. in Biological Sciences: Toxicological Biochemistry. She is a member of the UFFS research advisory committee and a member of the Biovitta Research Institute. She is currently the leader of the research group: Biological and Clinical Studies in Human Pathologies, professor of the postgraduate program in Biochemistry at UFSC, and postgraduate program in Biomedical Sciences by UFFS. She acts mainly on the following topics: oxidative stress, purinergic system, and human pathologies being a reviewer of several international journals and books.
